# Widespread Over-Expression of the X Chromosome in Sterile F_1_ Hybrid Mice

**DOI:** 10.1371/journal.pgen.1001148

**Published:** 2010-09-30

**Authors:** Jeffrey M. Good, Thomas Giger, Matthew D. Dean, Michael W. Nachman

**Affiliations:** 1 Department of Ecology and Evolutionary Biology, University of Arizona, Tucson, Arizona, United States of America; 2 Department of Evolutionary Genetics, Max Planck Institute for Evolutionary Anthropology, Leipzig, Germany; 3 Division of Biological Sciences, University of Montana, Missoula, Montana, United States of America; 4 Molecular and Computational Biology, University of Southern California, Los Angeles, California, United States of America; Stanford University, United States of America

## Abstract

The X chromosome often plays a central role in hybrid male sterility between species, but it is unclear if this reflects underlying regulatory incompatibilities. Here we combine phenotypic data with genome-wide expression data to directly associate aberrant expression patterns with hybrid male sterility between two species of mice. We used a reciprocal cross in which F_1_ males are sterile in one direction and fertile in the other direction, allowing us to associate expression differences with sterility rather than with other hybrid phenotypes. We found evidence of extensive over-expression of the X chromosome during spermatogenesis in sterile but not in fertile F_1_ hybrid males. Over-expression was most pronounced in genes that are normally expressed after meiosis, consistent with an X chromosome-wide disruption of expression during the later stages of spermatogenesis. This pattern was not a simple consequence of faster evolutionary divergence on the X chromosome, because X-linked expression was highly conserved between the two species. Thus, transcriptional regulation of the X chromosome during spermatogenesis appears particularly sensitive to evolutionary divergence between species. Overall, these data provide evidence for an underlying regulatory basis to reproductive isolation in house mice and underscore the importance of transcriptional regulation of the X chromosome to the evolution of hybrid male sterility.

## Author Summary

The X chromosome plays an important role in the development of reproductive isolation between species, but the basis for this has remained unclear. One possible explanation is that sperm development is sensitive to disruption of X-linked gene regulation. In mice, evidence linking abnormal gene expression on the X chromosome with reproductive isolation has been lacking until now. Here we use experimental crosses within and between species of mice and genome-wide expression data to identify aberrant expression patterns associated with hybrid male sterility. We observed chromosome-wide over-expression of the X chromosome during spermatogenesis in sterile hybrid males and developmentally localized this breakdown to an apparent disruption of X-inactivation. Collectively, these results highlight the importance of gene regulation to the evolution of reproductive isolation and support the hypothesis that improper expression of the X chromosome during spermatogenesis is an important mechanism contributing to the rapid evolution of hybrid male sterility.

## Introduction

The importance of proper gene regulation to the evolution of reproductive isolation between species is not well understood. Several studies have documented abnormal genome-wide patterns of expression in F_1_ hybrid offspring relative to their parental species [1]–[5]. However, two confounding factors make it difficult to determine the extent to which these data are directly relevant to the genetic basis of speciation. First, expression data from whole tissues reflect proportional transcript abundances across different cell types. Thus, genome-wide differences in hybrid expression could simply reflect quantitative differences in the cellular composition of tissues that manifest abnormal hybrid phenotypes [6], rather than true expression differences between cells. Second, many studies have focused on divergent crosses that produce severe F_1_ hybrid incompatibility phenotypes that uniformly affect a given sex. Without variability in F_1_ sterility, it is difficult to establish a causal relationship between reproductively isolating phenotypes and general expression patterns on a hybrid genomic background.

The X chromosome often plays a central role in the genetic underpinnings of reproductive isolation [7]. Hybrid inviability and sterility typically arise due to incompatible epistatic interactions between divergent genes [7]–[9]. Deleterious recessive incompatibilities are exposed on the X chromosome, but not the autosomes, of F_1_ hybrid males. This dominance-based model [10] provides a simple genetic explanation for the ubiquitous evolutionary pattern that hybrid inviability or sterility overwhelmingly afflicts the heterogametic sex first (i.e., Haldane's rule [11]). However, it does not explain why hybrid male sterility evolves much faster than male inviability [12]–[16]. Male sterility evolves particularly quickly on the X chromosome (i.e., the large X-effect [7], [17] for male sterility), which has been shown in *Drosophila* to accumulate a higher density of recessive mutations causing hybrid male sterility relative to the autosomes [14]–[16]. There are several evolutionary hypotheses to explain the rapid development of X-linked sterility, including more frequent positive selection on the X chromosome because of the immediate exposure of beneficial recessive mutations [18]–[20], recurrent genetic conflict over the meiotic transmission of the sex chromosomes [21]–[25], and rampant gene movement onto and off of the X chromosome [26]. None of these hypotheses are mutually exclusive and all plausibly contribute to the rapid evolution of hybrid male sterility.

Rapid X-linked evolution notwithstanding, the importance of the X chromosome for hybrid male sterility is surprising given that spermatogenic genes tend to be underrepresented on the X chromosome [27]–[29]. A possible mechanistic explanation for this discrepancy is that spermatogenesis may be particularly sensitive to disruption of gene expression on the X chromosome [16], [30]–[32]. In mammals [33], flies [34], and nematodes [35], transcription on the X chromosome is silenced during part of spermatogenesis, resulting in an under-representation of X-linked spermatogenic genes [27], [29], [36]. In mice, the X chromosome is inactivated at the pachytene stage of meiosis (i.e., meiotic sex chromosome inactivation or MSCI) when homologous autosomes synapse [37], [38]. Most of the X chromosome remains transcriptionally inactive for the duration of spermatogenesis (postmeiotic sex chromosome repression or PMSR) save a relatively small subset of postmeiotically expressed genes [39]. Mutations that disrupt synaptic pairing of autosomes can disrupt MSCI and PMSR, often resulting in male-limited sterility [40]–[42]. If MSCI and/or PMSR are also sensitive to evolutionary divergence between closely related species then disruption of X-inactivation during spermatogenesis may provide a general molecular basis for the large X-effect and the rapid evolution of hybrid male sterility [30]–[32].

Two closely related lineages of house mice, *Mus musculus* and *M. domesticus,* provide a powerful system for studying the role of gene regulatory divergence in speciation. The two species are recently diverged (∼500 KYA; [43]) and form a narrow hybrid zone across Europe. Laboratory crosses between *M. domesticus* and *M. musculus* often yield fertile females and sterile males [44]. The spermatogenic status of F_1_ males ranges from normal to complete meiotic arrest [31] or dramatic reductions in postmeiotic cells [44]. Two factors contribute to variation in F_1_ hybrid male sterility. First, multiple sets of epistatic incompatibilities are involved in spermatogenic failure [31], [44], including one or more X-autosome interactions that result in asymmetric sterility in some reciprocal crosses [44], [45]. All asymmetric crosses described so far yield sterile hybrid males when the maternal line is *M. musculus,* and introgression of the *M. musculus* X chromosome causes male sterility on a *M. domesticus* genetic background [46], [47]. Second, multiple autosomal incompatibilities are polymorphic within *M. musculus* and *M. domesticus* [45], [47]–[49]. Thus, F_1_ hybrid male fertility depends critically on both the direction of the cross and the genotype of the parental species.

One of the polymorphic incompatibilities, *Hst1*, has recently been localized to a single autosomal gene, PR-domain 9 or *Prdm9* [50]. *Prdm9* is involved in histone methylation [51] and causes aberrant expression of several interacting genes in sterile hybrid males [50]. Two previous studies [5], [52] have interrogated the evolution of gene expression between *M. musculus*, *M. domesticus*, and *M. castaneus* (another closely related species). The results of these studies were somewhat conflicting, with testis showing a clear excess of expression divergence between *M. musculus* and *M. domesticus* relative to brain or liver in only one of the experiments (i.e., [5]). This experiment also evaluated F_1_ hybrid male expression for two reciprocal crosses (*M. domesticus* and *M. musculus; M. castaneus* and *M. musculus*) [5]. F_1_ expression patterns were largely additive in most tissues and crosses; however, males from one cross (female *M. musculus* x male *M. castaneus*) showed an excess of mis-expressed transcripts in testis. The relevance of these data to mouse speciation remains unclear because sterility factors are polymorphic within house mice [45], [47]–[49] and male fertility phenotypes were not measured in this experiment.

Here we evaluate the role of gene expression in mouse speciation by using a reciprocal cross between *M. domesticus* and *M. musculus* that results in asymmetric hybrid male sterility. We directly associate aberrant expression patterns with hybrid male sterility by contrasting genome-wide expression data for both species with data from fertile and sterile F_1_ hybrids. Previously published phenotypic data from sterile hybrid males [44] were used to generate simple qualitative predictions for expected expression differences due to changes in the cellular composition of sterile hybrid testis. We then considered these predictions in the context of detailed information on the developmental timing of gene expression during spermatogenesis [39], [53].

## Results/Discussion

### Experimental design

**Figure 1:**
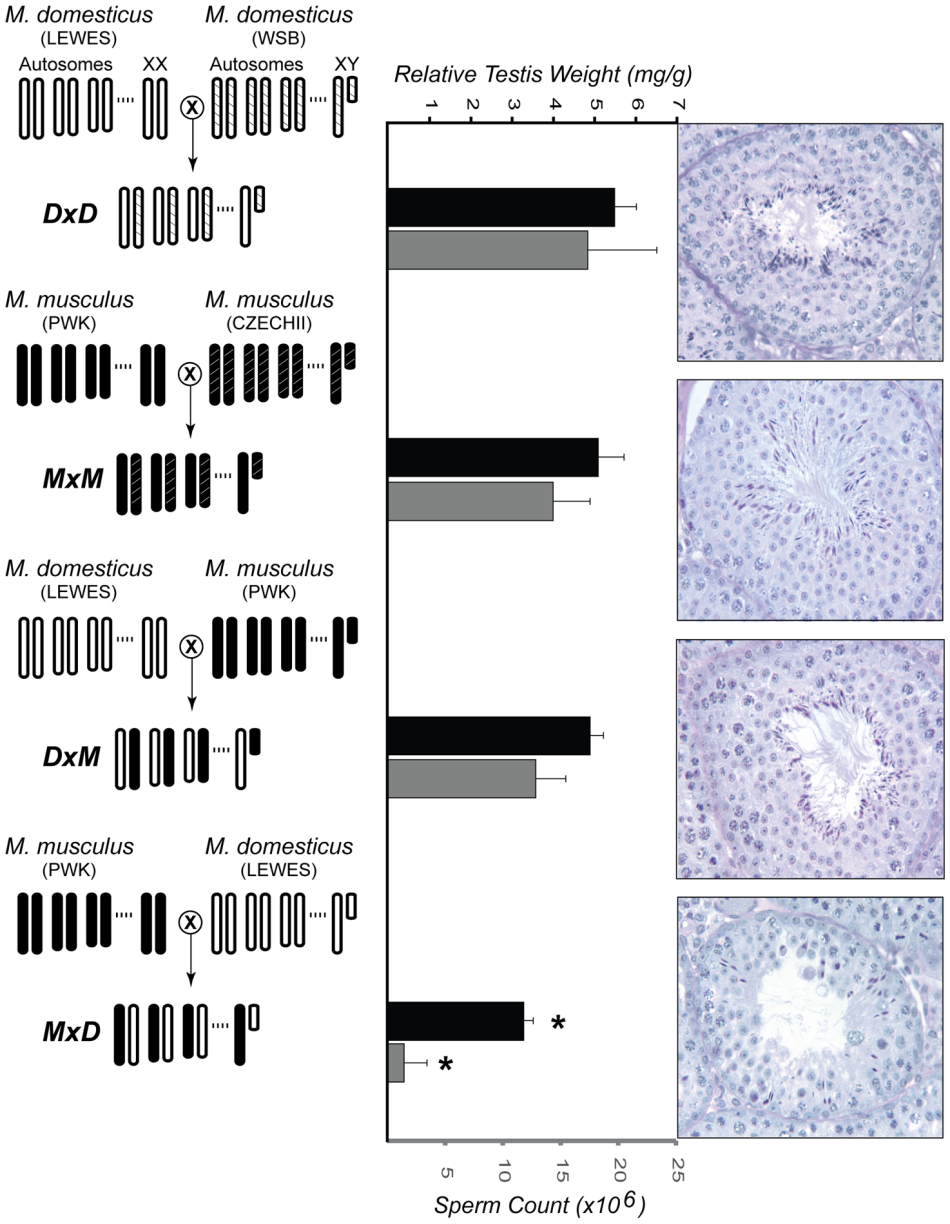
Experimental design and male reproductive phenotypes. The crossing designs used to generate F_1_ males from two intraspecific crosses (*DxD* and *MxM*) and two interspecific crosses (*DxM* and *MxD*) are shown on the left. For each cross, the genotype for three autosomes and both sex chromosomes are given. Light-colored chromosomes (white and white-hatched) are from wild-derived strains of *M. domesticus* (LEWES/EiJ, WSB/EiJ) and dark-colored chromosomes (black and black-hatched) are from wild-derived strains of *M. musculus* (PWK/PhJ, CZECHII/EiJ). Previously published estimates [44] of relative testis weights (standardized for body weight) and sperm counts are given for males from each cross, in black and gray bars, respectively. Error bars indicate one standard deviation. (*) Hybrid *MxD* males were significantly reduced for both characters when compared to *MxM* and *DxD* males (pooled, Wilcoxon rank sum, P<0.01). For each genotype, representative histological cross-sections of a single seminiferous tubule are shown on the right. The first three genotypes showed normal progression of spermatogenesis, while the *MxD* males showed diminished numbers of germ cells overall, poor organization of the seminiferous epithelium, and a large reduction in the number of postmeiotic cells.

The current work builds upon a previous study examining hybrid male sterility [44]. Previously, all eight pairwise interspecific crosses were performed between two wild-derived strains of *M. domesticus* (LEWES/EiJ, WSB/EiJ) and two wild-derived strains of *M. musculus* (PWK/PhJ, CZECHII/EiJ). F_1_ hybrid males from reciprocal interspecific crosses between CZECHII/EiJ and either strain of *M. domesticus* had small testis that produced few or no mature sperm. In contrast, males from crosses involving PWK/PhJ were only sterile when PWK/PhJ was the maternal strain (Figure 1).

Focusing on this latter asymmetric cross involving PWK/PhJ, we interrogated expression levels of ∼39,000 transcripts using Affymetrix Mouse Genome 430 2.0 GeneChips. For each of four genotypes (Figure 1), we examined expression levels of RNA isolated from whole testis in three 60-day old males resulting in 12 microarray experiments. This cross design was chosen specifically to evaluate the expression of a single *M. musculus* and *M. domesticus* X chromosome on both con- and heterospecific F_1_ backgrounds. Crosses within each species were performed to avoid confounding expression and phenotypic differences within and between species with differences between inbred and F_1_ genotypes. This design provides two important contrasts for evaluating the contribution of expression differences to reproductive isolation. First, comparison of testis expression levels within *M. musculus* (hereafter *MxM*) and within *M. domesticus* (hereafter *DxD*) males allows for the identification of genes with divergent expression levels between the species. Second, comparison between sterile hybrid males (hereafter *MxD*, maternal strain first) and all other males, including the fertile reciprocal hybrid (hereafter *DxM*), provides a direct contrast between normal and sterile males.

### Strong conservation of testis expression between species on the X chromosome

Affymetrix 430 2.0 GeneChips were designed from the genome of the laboratory mouse C57BL/B6, which is largely of *M. domesticus* origin [54]. To help reduce the influence of probe mismatch, we incorporated probe performance into our analysis by down-weighting probes with high technical variance and only including genes that were detected in all samples based on Wilcoxon signed rank tests (P*<*0.01) between perfect versus mismatch signals. Of the 6,998 genes detected in all 12 samples (Table S1), 2,065 were significantly different between *M. musculus* and *M. domesticus* (P<0.05, pairwise t-tests). 1,435 of these genes remained significant at an estimated false discovery rate (FDR) of 5% (P<0.02364, pairwise t-tests).

The cellular composition of testis is highly heterogeneous, including populations of both somatic and germ line cells. Therefore, expression data collected from whole testis can be strongly influenced by the underlying cellular composition. We used published expression data [53] to identify groups of genes that show the greatest level of induction in somatic (Sertoli cells), mitotic (spermatogonia), meiotic (spermatocytes), or postmeiotic (round spermatids) cells. Of the 1,435 genes with significantly different expression between the species (FDR<0.05), 712 genes could be associated with a particular cell type. For these genes, loci with significantly higher expression in *M. domesticus* were enriched for meiotic genes but under-represented among mitotic genes (Table 1). Expression differences were not biased with respect to postmeiotic genes, suggesting that this difference does not reflect a simple shift in the onset of spermatogenesis (i.e., later development in *M. musculus*). Rather, it appears that evolutionary differences between the species are enriched to particular developmental time-points (i.e., meiosis). However, it is also possible that a subtle shift in the overall cellular composition of the testis has evolved between the species. Note that a slight majority of these differences resulted from transcripts that were more highly expressed in *M. musculus* (55%). These data suggest that probe effects due to evolutionary divergence are not a major factor in our analysis because probe mismatches to *M. musculus* should bias our results towards transcripts appearing more highly expressed in *M. domesticus*.

**Table 1:** Expression differences between *M. domesticus* and *M. musculus* across spermatogenic cell types.

	Autosomes			X chromosome		
	observed	expected*	P	observed	expected*	P
**All genes (N = 712)**						
Somatic	80	85.9	1	5	3.0	0.6300
Mitotic	172	220.2	0.0003	6	9.2	0.4345
Meiotic	284	233.9	0.0003	-	-	
Postmeiotic	159	155.0	1	6	4.7	1
**Higher expression in ** ***M. domesticus*** ** (N = 320)**						
Somatic	31	38.8	0.7937	1	1.1	1
Mitotic	52	99.5	<0.0001	2	3.3	1
Meiotic	154	105.7	<0.0001	-	-	-
Postmeiotic	77	70.0	1	3	1.7	1
**Higher expression in ** ***M. musculus*** ** (N = 392)**						
Somatic	49	47.1	1	4	2.0	0.1182
Mitotic	120	120.7	1	4	6.0	0.2455
Meiotic	130	128.2	1	-	-	-
Postmeiotic	82	85.0	1	3	3.1	1

*Expectations were generated independently for the X chromosome and the autosomes based on the observed distributions of expressed genes in a given cell type and tested with a Bonferroni-corrected binomial distribution.

Dashes (-) denote that no meiotic genes were observed on the X chromosome.

**Figure 2:**
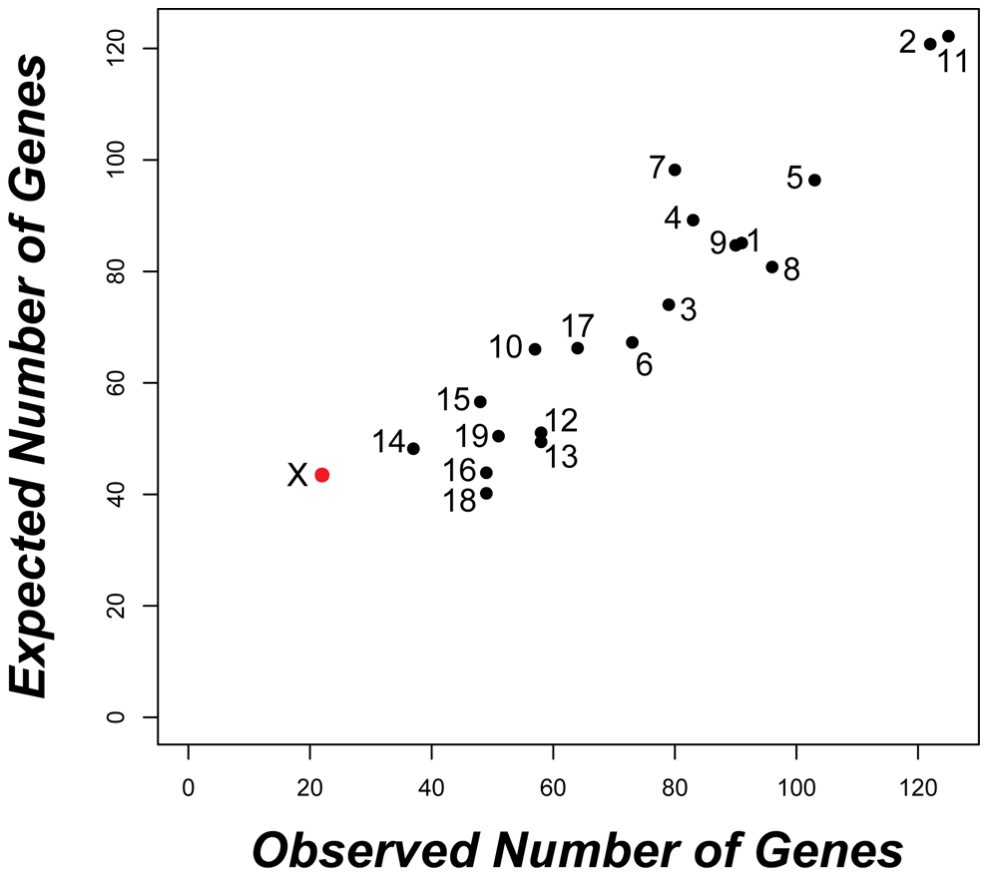
Strong conservation of testis expression on the X chromosome between species. The observed versus expected distribution of the 1,435 genes with significantly different expression between *M. musculus* and *M. domesticus* (P<0.02364; FDR<0.05) is given for each chromosome. Only the X chromosome (red) showed a significant deviation (22 observed versus 43.5 expected; Bonferroni-corrected P<0.003) based on chromosome-wise hypergeometric tests.

Autosomal genes with divergent expression between species were distributed as expected given the genomic location of probes on the array (Figure 2). In contrast, we found half as many significant differences as expected on the X chromosome (22 observed versus 43.5 expected; Bonferroni-corrected P<0.003). The same under-representation of differences on the X chromosome was also obtained for the larger set of 2,065 genes (35 X-linked observed versus 63 expected; Bonferroni-corrected P<0.001) identified using a non-FDR corrected cutoff for the pairwise t-tests (P<0.05). These results are seemingly at odds with the prediction that the X chromosome may be disproportionately involved in adaptive evolution [18]. Although empirical evidence for faster X-linked evolution has been mixed [32], numerous studies have reported higher levels of protein divergence [55]–[57], a higher incidence of positive selection [56], [58], and an over-representation of certain classes of male reproductive genes [29], [59] on the X chromosome. We found that X-linked testis-expressed genes also show a significantly higher rate of protein evolution than autosomal testis-expressed genes (Wilcoxon signed rank P<0.0001 for *dN/dS* pairwise comparison versus orthologous rat genes; X chromosome, N = 152 genes, mean = 0.247, median = 0.160; autosomal *dN/dS*, N = 5,611 genes, mean = 0.165, median = 0.112). Thus, contrary to considerable evidence for rapid protein evolution on the X chromosome, our results demonstrate that testis gene expression on the X chromosome is actually more highly conserved between species of mice. Testis expression is significantly enriched for mitotic expression on the X chromosome (Table S2) and MSCI selects against expression during meiosis [29]. These developmental constraints also appear to limit X-linked expression divergence between species.

### Widespread mis-expression of the X chromosome in sterile hybrid males

**Figure 3:**
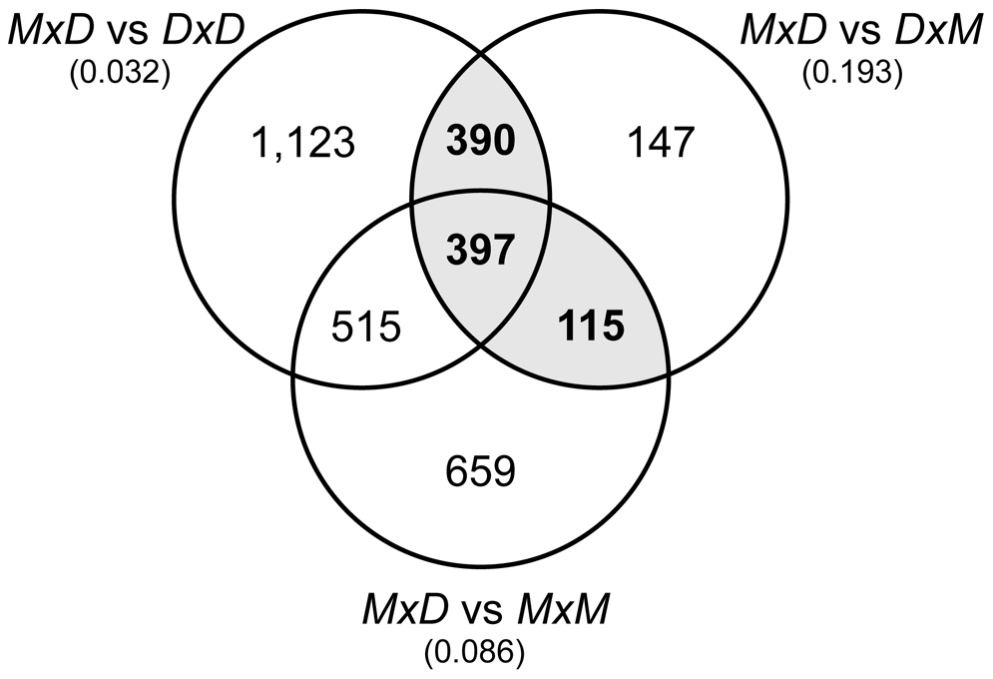
Overlap of pairwise expression differences between sterile and fertile mice. The Venn diagram gives the numbers of genes with significantly different expression (P<0.05) for the three pairwise contrasts between sterile *MxD* mice and the three fertile mouse genotypes (*DxM, MxM, DxD*). The estimated FDR for each comparison is given in parentheses. There were 902 genes that were significantly different between the reciprocal hybrids and at least one of the parental lines (gray shading).

To associate expression divergence with reproductive isolation, we employed a hierarchical approach to define a conservative set of sterility-correlated genes. First, we contrasted each of the three fertile genotypes (*MxM*, *DxD*, *DxM*) with the *MxD* sterile F_1_ hybrid mice and identified all genes with significantly different expression between groups based on gene-by-gene t-tests (P<0.05; Figure 3). The estimated FDR among significant differences identified in these three pairwise contrasts ranged from 3.2% (*MxD* vs. *DxD*) to 19.3% (*MxD* vs. *DxM*). To help reduce the global FDR while enriching for expression differences directly correlated with the sterility phenotype, we focused only on the 902 genes that were significantly different between the reciprocal hybrids and at least one of the parental lines (Figure 3). We refer to these 902 genes as “sterility-correlated genes”. The autosomal distribution of sterility-correlated genes did not deviate from random expectations (Figure S1). However, opposite to what was observed between species, we detected a ∼three-fold enrichment of sterility-correlated genes on the X chromosome (81 observed versus 27.3 expected, Bonferroni-corrected P<0.0001). Importantly, an approximately three-fold enrichment of sterility-correlated genes on the X chromosome was consistently observed across different operational definitions of “sterility-correlated”: the 1,049 genes differing between sterile *MxD* males and fertile *DxM* males (94 observed versus 31.8 expected, Bonferroni-corrected P<0.0001), the 397 genes different in all three fertile vs. sterile pairwise contrasts (43 observed versus 12 expected, Bonferroni-corrected P<0.0001), and the 181 genes different between all three fertile vs. sterile pairwise contrasts and not different between all three fertile genotypes (21 observed versus 5.5 expected, Bonferroni-corrected P<0.0001). Likewise, we also observed a strong global enrichment of sterility-correlated genes on the X chromosome (46 observed versus 6.8 expected, Bonferroni-corrected P<0.0001) when using a more conservative cutoff (P<0.01; estimated FDR 0.5–7.5%) in our pairwise t-tests (Figure S2).

Histological analyses show that sterile *MxD* males have a dramatic reduction in the number of postmeiotic cells (Figure 1, [44]). Thus, postmeiotic cells comprise a smaller proportion of the overall cellular composition of testis in sterile *MxD* testis when compared to fertile males with normal spermatogenesis. Therefore, genes expressed late in spermatogenesis would be expected to show lower expression levels in these sterile males, even if transcript abundances *per cell* were equivalent. Because we are measuring transcript levels from a fixed amount of RNA extracted from whole testis, it follows that transcripts from mitotic cells would be proportionally more common in sterile males. This simple qualitative model, hereafter referred to as the “cellular composition hypothesis”, predicts that mitotic genes should appear to be over-expressed while postmeiotic genes should show lower expression in sterile hybrid males. For example, postmeiotic cells comprise ∼85% of the total cellular content of adult testis [60]. If postmeiotic cells only comprised 55% of the cells in the testis of sterile F_1_ males then we would expect an apparent reduction in postmeiotic expression to be accompanied by a three-fold proportional increase in the relative abundance of non-postmeiotic transcripts (i.e., an increase from 15% to 45% of total testis cellular composition). Only genes with higher postmeiotic expression and lower mitotic expression in sterile males are not confounded by cellular composition and potentially reflect true expression differences. Moreover, such differences should be highly conservative because the skew in cellular composition should reduce our power to detect true expression differences. In particular, the skew in cellular composition will lead to an underestimate of the magnitude of differences that is proportional to the underlying difference in relative abundance of the relevant cell type.

**Figure 4:**
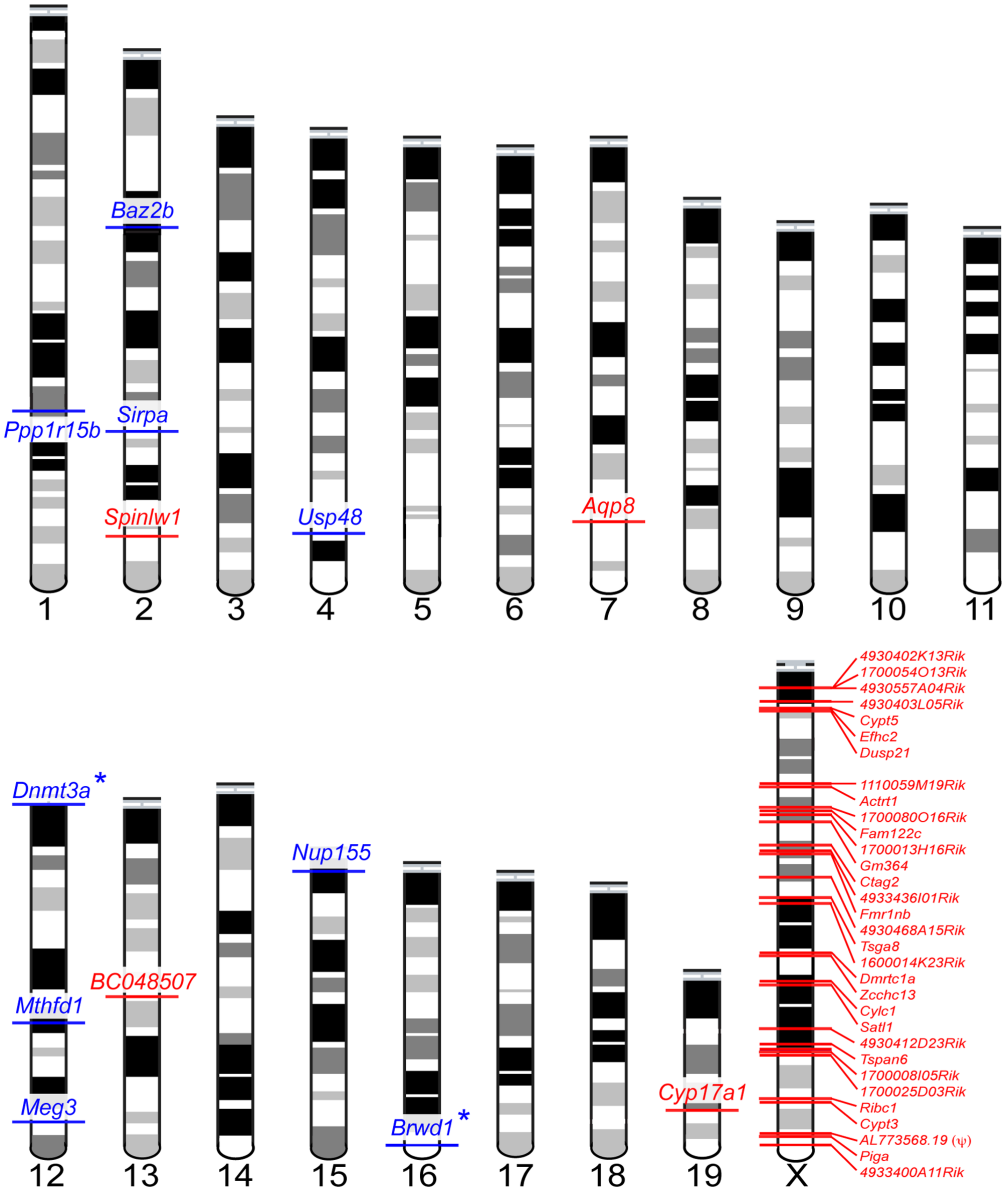
Forty-five sterility-correlated genes with patterns of expression robust to differences in testis cellular composition between fertile and sterile males. Postmeiotic genes over-expressed in sterile males are shown in red, mitotic genes under-expressed in sterile males are shown in blue. Autosomal loci with known male sterility knockout phenotypes are indicated with an (*). One locus, AL773568.19(ψ), is a transcribed pseudogene.

To evaluate our data in the context of the cellular composition hypothesis, we first binned the 902 sterility-correlated genes into three groups: genes with higher expression in sterile *MxD* mice, genes with lower expression in sterile *MxD* mice, and genes with intermediate expression in sterile *MxD* mice. We then identified 607 sterility-correlated genes that could be associated with one of four spermatogenic cell types [53]. Overall, postmeiotic genes were highly over-represented among sterility-correlated genes and there were many fewer meiotic genes than expected by chance (Table 2). However, this global pattern masks key differences in gene expression between the X chromosome and the autosomes. Autosomal sterility-correlated genes closely followed the predictions of the cellular composition hypothesis with most postmeiotic genes showing lower expression (180 of 211, ∼85%) and most mitotic genes showing higher expression (151 of 184, ∼82%) in sterile *MxD* males. These differences are confounded by the skewed cellular composition of the sterile versus fertile males and thus may not reflect true differences in expression. In stark contrast, most of the 902 sterility-correlated genes on the X chromosome were over-expressed in sterile *MxD* males (∼93% or 75 of 81). Thus, simple differences in the cellular composition of sterile and fertile hybrid males do not explain a majority of mis-expressed genes on the X chromosome. We also observed an almost 2-fold increase in over-expressed postmeiotic genes on the X chromosome (32 observed, 18 expected; Table 2). Strikingly, the X chromosome harbors only ∼6% of the postmeiotic genes in our dataset (48 of 806) yet 89% of the postmeiotic genes (32 of 36) that were over-expressed in sterile *MxD* males were X-linked. Overall, there were 45 sterility-correlated genes that could not be explained by simple differences in the cellular composition of testis from sterile and fertile males (higher postmeiotic, lower mitotic expression in sterile males, Table S3); thirty-two of these were over-expressed postmeiotic genes on the X chromosome (Figure 4).

**Table 2:** Expression of sterility-correlated genes across spermatogenic cell types.

	Autosomes			X chromosome		
	observed	expected*	P	observed	expected*	P
**All genes (N = 607)** †						
Somatic	55	66.5	0.5964	9	12.4	1
Mitotic	184	170.5	0.8445	26	37.5	0.0222
Meiotic	88	181.1	<0.0001	-	-	-
Postmeiotic	211	120.0	<0.0001	34	19.1	0.0006
**Higher expression in sterile ** ***MxD*** ** males (N = 260)** ‡						
Somatic	34	24.1	0.1519	8	11.6	0.9903
Mitotic	151	61.8	<0.0001	25	35.3	0.0369
Meiotic	6	65.6	<0.0001	-	-	-
Postmeiotic	4	43.5	<0.0001	32	18.0	0.0008
**Lower expression in sterile ** ***MxD*** ** males (N = 255)** ‡						
Somatic	11	31.4	<0.0001	0	0.2	1
Mitotic	9	80.5	<0.0001	0	0.5	1
Meiotic	54	85.5	0.0032	-	-	-
Postmeiotic	180	56.6	<0.0001	1	0.3	0.8323
**Intermediate expression in sterile ** ***MxD*** ** males (N = 92)** ‡						
Somatic	10	11	1	1	0.5	1
Mitotic	24	28.2	1	1	1.6	1
Meiotic	28	30	1	-	-	-
Postmeiotic	27	19.8	0.2983	1	0.8	1

*Expectations were generated independently for the X chromosome and the autosomes based on the observed distributions of expressed genes in a given cell type and tested with a Bonferroni-corrected binomial distribution.

**†:** Represents the subset of sterility-correlated genes that could be associated with cell types, excluding one gene on the Y chromosome.

**‡:** Genes with higher, lower, or intermediate average expression in sterile *MD* males relative to the average expression level in each of the three fertile genotypes.

Dashes (-) denote that no meiotic genes were observed on the X chromosome.

**Figure 5:**
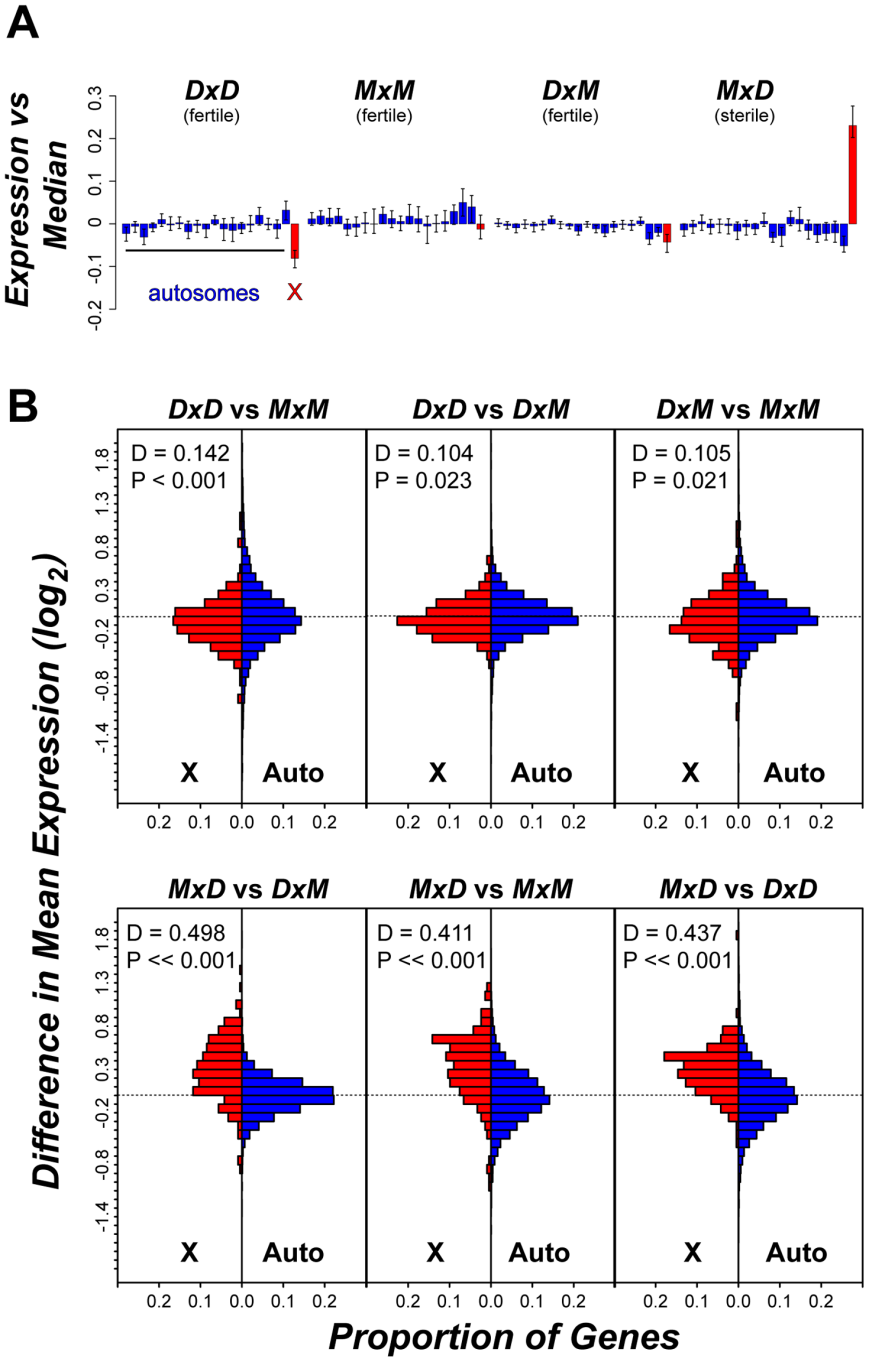
Over-expression of the X chromosome in sterile *MxD* hybrid mice. (A) Average per chromosome deviation (log_2_ scale) for each genotype versus the median per gene expression across all 12 males. The 19 autosomes (blue) are presented sequentially with the X chromosome (red). (B) Distribution of pairwise expression differences for the X chromosome versus the autosomes. For each pairwise comparison, the proportional distribution of gene-by-gene differences in mean expression (log_2_) is shown for the X chromosome (red) and the autosomes (blue) with results from a Kolmogorov-Smirnoff test.

Two patterns indicate that the signature of higher X-linked expression in sterile *MxD* males is a chromosome-wide phenomenon. First, the 32 postmeiotic genes over-expressed in sterile *MxD* males were distributed across the majority of the X chromosome (8.7–166.2 Mb, Table S3, Figure 4). Second, over-expression of the *MxD* X chromosome was also apparent when considering the per chromosome deviation of all 6,998 expressed genes (Figure 5). Genes on the X chromosome of the sterile *MxD* mice showed a mean increase of 17% compared to the per gene median expression level across all males (Figure 5A). In each of the six pairwise comparisons, the distribution of expression differences was significantly different between the X chromosome and the pooled autosomes (Figure 5B). However, the two chromosomal groups were similar and centered near zero for the three comparisons between fertile genotypes (Figure 5B, top panel), while the three contrasts involving sterile males all showed a dramatic shift towards higher X-linked expression in sterile *MxD* males (Figure 5B, bottom panel).

The observed over-expression of the X chromosome was also not merely a consequence of probe-induced artifacts. Higher expression of the X chromosome in sterile *MxD* mice is opposite of what would be expected if our results were strongly influenced by reduced probe affinity due to divergence from the *M. domesticus*-derived microarray, because the X chromosome in the sterile males is of *M. musculus* origin. More specifically, the fertile *MxM* mice and the sterile *MxD* mice share the same hemizygous *M. musculus* X chromosome and thus can be used to further evaluate X-linked expression on con- and heterospecific F_1_ backgrounds, independent of X-linked probe effects. A recent study [39] provided a detailed account of cell type specific expression patterns on the X chromosome, which is independent of the testis cell type associations we used above [53]. This work identified five general patterns of X-linked spermatogenic expression in mice: (1) expressed primarily in mitotic cells and repressed in meiotic and postmeiotic cells; (2) expressed in mitotic cells, repressed in meiotic cells, and expressed in postmeiotic cells; (3) expressed primarily in postmeiotic cells; (4) variable expression; (5) repressed in all cells. Associating these X-specific expression groups with our testis expression data from *MxM* and *MxD* males, we again found that most genes (154 of 180) showed higher average expression on the *M. musculus* X chromosome of *MxD* males, including 27 of 31 genes expressed primarily in postmeiotic cells (Figure S3). The strong tendency for X-linked genes to show higher expression in *MxD* males is in contrast to the slight bias (52% of genes) in the opposite direction for autosomal genes in this pairwise comparison.

By incorporating a detailed understanding of the sterility phenotype with information on the progression of gene expression during spermatogenesis, we were able to establish a striking association between F_1_ sterility and mis-regulation of the X chromosome that appears to be independent of differences in the cellular composition of the testis of sterile and fertile mice. The large role of the X chromosome also does not appear to be a direct consequence of greater evolutionary divergence for X-linked gene expression because X chromosome expression appears exceptionally conserved between species (Figure 2). Finally, the large role of the X chromosome inferred from this study is consistent with previous mapping results using these same strains, which showed that many loci distributed along the entire *M. musculus* X chromosome play an important role in hybrid male sterility [47]. Therefore, these data support the hypothesis that spermatogenic gene regulation on the X chromosome is particularly sensitive to incompatible interactions between the divergent genomes of *M. musculus* and *M. domesticus.* With the current data, we cannot determine whether higher expression of the X chromosome results from a failure of MSCI or abnormally high postmeiotic transcription. However, we did not observe a pachytene arrest during meiosis I [44] as might be expected given a complete failure of MSCI. Additional fine-scale examination of transcription on the X chromosome during the developmental progression of spermatogenesis should help resolve the exact timing and mechanism underlying this pattern.

### X-inactivation, mis-expressed autosomal genes, and epigenetic regulation

Intrinsic hybrid incompatibilities typically arise due to epistatic interactions among divergent genes [7]–[9]. In our experiment, over-expression of the X chromosome only occurred on the *MxD* hybrid genomic background and thus likely involves one or more epistatic interactions between the *M. musculus* X chromosome and loci on the *M. domesticus* Y and/or the autosomes. Differences in the cellular composition of the testes of sterile and fertile mice may mask many differentially expressed autosomal transcripts between the sterile and fertile mice. Nevertheless, inspection of the 13 autosomal sterility-correlated genes with expression patterns that should be robust to differences in cellular composition (Figure 4; Table S3) revealed two compelling candidates for genes contributing to regulatory incompatibilities. First, *DNA methyltransferase 3A* (*Dnmt3a*) showed significantly reduced expression in sterile *MxD* males versus all three fertile genotypes (all P<0.021). *Dnmt3a* is essential for *de novo* DNA methylation [61] and conditional knockouts of *Dnmt3a* cause complete spermatogenic arrest characterized by a failure of germ cells to develop past round spermatids [62]. Under-expression of *Dnmt3a*, given its general role as a repressor of transcription via DNA methylation, and the global over-expression of X chromosome in our sterile mice (Figure 5), together raise the possibility of a direct connection between *Dnmt3a* and the failure of MSCI or PMSR. *Dnmt3a* is a direct negative regulator of *Xist* [63], which is required for X-inactivation in females [64]. In males, *Xist* is exclusively expressed in testis coincident with the onset of MSCI [65]. However, MSCI proceeds normally in males with a disrupted copy of *Xist* [66], suggesting X-inactivation proceeds through sex-specific mechanisms [41]. Thus, while *Dnmt3a* is essential for spermatogenesis, its underlying role in MSCI and PMSR remains unresolved.

Second, the transcription factor *Brwd1* (Bromodomain and WD repeat domain containing 1) also showed significantly reduced expression in sterile hybrid *MxD* males versus the three other fertile genotypes (all P<0.005). *Brwd1* is thought to influence transcriptional regulation and chromatin remodeling during spermatogenesis and oogenesis [67]. Mice homozygous for a null mutant of *Brwd1* show both male and female sterility [67]. In testis, *Brwd1* is most highly expressed in spermatogonia [53], yet disruption of *Brwd1* in males results primarily in postmeiotic disruption of spermatogenesis, including dramatic reduction in postmeiotic spermatocytes, low epididymal sperm counts, abnormal sperm head morphology, and poor motility [67]. These phenotypes are qualitatively similar to those found in both sterile *MxD* males (Figure 1; [44]) and male-sterile strains of *M. domesticus* (LEWES) consomic for portions of the *M. musculus* (PWK) X chromosome [47].

In addition to *Dnmt3a* and *Brwd1,* a histone methyltransferase gene on chromosome 17, *Prdm9*, was recently determined to be involved in hybrid male sterility between *M. musculus* and *M. domesticus* [50], marking the first discovery of a hybrid sterility locus in a vertebrate. *Prdm9* expression was not detected in any of the males in our experiment, suggesting expression levels were beyond the limits of microarray detection. Nevertheless, *Prdm9* warrants further consideration in the context of abnormal F_1_ gene expression. Null mutants of *Prdm9* disrupt homologous chromosome pairing and sex body formation during meiosis, resulting in male and female sterility [51]. Crosses between female *M. musculus* (PWD) and male C57BL/B6 (a laboratory strain predominantly of *M. domesticus* origin [54]) result in complete meiotic arrest of hybrid males due to an epistatic interaction between *Prdm9* and multiple unidentified autosomal and X-linked factors [46], [68]. However, genotypic data suggest that *Prdm9* is not involved in hybrid sterility in our experiment. *Prdm9* is polymorphic for fertile and sterile alleles in laboratory strains of mice (i.e., ∼*M. domesticus*), and hybrid sterility only ensues when both sterility alleles are present [31]. The only protein-coding difference between sterile and fertile *M. domesticus Prdm9* alleles is variation in the number of C-terminal C_2_H_2_ zinc-finger repeats [50]. We found that fertility-associated *Prdm9* length variants also segregate between wild-derived strains of *M. domesticus* (LEWES, fertile allele; WSB, sterile allele) yet both of these strains produce sterile hybrid males with largely postmeiotic abnormalities when crossed with female *M. musculus* (PWK) [44]. Moreover, the sterile *MxD* males carry the *Prdm9* length variant associated with fertility. Thus, if *Prdm9* was involved in sterility of *MxD* males, it would require different allelic combinations than previously described [50].

### The role of X-inactivation and epigenetic gene regulation in the evolution of hybrid male sterility

The global patterns we have described argue that disruption of gene regulation plays an important role in house mouse speciation. Several lines of evidence suggest that the X chromosome plays a large role in reproductive isolation in house mice, and that the genetic basis of this isolation is reasonably complex [47]. Three studies have attempted to dissect the genetic basis of hybrid sterility through introgression of the *M. musculus* X chromosome on to largely *M. domesticus* genomic backgrounds [46], [47], [69]. All three studies identified multiple QTL of large effect associated with male sterility spanning the X chromosome, but finer-scale localization of individual loci has thus far proven elusive. Interestingly, previous crosses with these same strains revealed a near additive effect of many loci along the entire X chromosome contributing to hybrid male sterility [47]. That observation, together with the genomic distribution of expression differences presented here, raises the possibility that sterility in these mice largely reflects the effects of disrupted transcriptional regulation of the X chromosome on a hybrid genomic background. This hypothesis predicts that the *M. musculus* X chromosome contains regulatory sequences along much of its length that do not interact properly with one or more *M. domesticus* autosomal loci. Chromosome-wide disruption of epigenetic silencing could also help explain an overall reduction in X-linked relative to autosomal gene flow between *M. musculus* X and *M. domesticus* observed in the European hybrid zone [70]–[73].

The X chromosome often plays a central role in speciation but the evolutionary basis for this has remained unclear. Several hypotheses, including faster evolution of the X chromosome [18] and an inherent sensitivity of spermatogenesis to disruption of X-linked gene regulation [30], [31], have been proposed to explain this phenomenon [32]. Our data provide empirical support for a regulatory basis to speciation in house mice and establish the importance of transcriptional regulation of the X chromosome in the evolution of hybrid male sterility, as originally proposed over 35 years ago [30]. Failure of MSCI may also play an important role in *Drosophila* speciation [32], where the X chromosome is enriched for over-expressed transcripts in testis of some sterile males [4]. In mammals, MSCI has long been argued as a critical check-point in male meiosis [74], [75] and failure of X-inactivation has been suggested to be an important cause of male sterility in humans and mice [41], [75]. In turn, disruption of X-inactivation may also prove to be an important mechanism contributing to two of the most general patterns in speciation genetics: Haldane's rule [11] and the disproportionately large effect of the X chromosome in hybrid male sterility [7], [17].

## Methods

### Ethics statement

Mice were maintained at the University of Arizona Central Animal Facility following Institutional Animal Care and Use Committee (IACUC) regulations.

### Strains, animal husbandry, and male reproductive phenotypes

All breeding colonies were established using individuals purchased from the Jackson Laboratory (Bar Harbor, ME). LEWES/EiJ and WSB/EiJ were originally derived from natural populations of *M. domesticus* in eastern North America and the *M. musculus* strains CZECHII/EiJ and PWK/PhJ were isolated from different localities within the Czech Republic. After weaning, male offspring were housed in sibling groups until 40 days postpartum, and then caged singly until being sacrificed at 60 days old. We collected data for several male reproductive phenotypes including testis weight, sperm count, sperm motility, seminal vesicle weight, testis histology, and fecundity. A detailed description of these data, including experimental protocols, has been published previously [44].

### Sample preparation and microarray processing

Immediately after males were euthanized, testes were dissected and cross-sectioned, placed in RNA*later* (Ambion, Inc., Austin, TX), and archived at −80C. We extracted total RNA from whole testis using an RNeasy Midi kit (QIAGEN Inc., Valencia, CA). RNA sample quality and quantification was determined with an RNA Nano LabChip on an Agilent Bioanalyzer 2100 (Santa Clara, CA). Only samples with an RNA integrity number of 10 were used. Biotinylated complementary DNA was generated from 5 µg of total RNA and hybridized to the Affymetrix Mouse Genome 430 2.0 array (Santa Clara, CA). Sample quality control and microarray processing was performed following the manufacturer's instructions by the Genomics Shared Service at the University of Arizona. In order to estimate the between chip experimental variability, we followed the standard protocol of spiking in transcripts for three genes from the biotin synthesis pathway in *E. coli* (BioB, BioC, BioD) and one transcript from the recombinase gene from bacteriophage P1 (cre) as hybridization controls. For all of these transcripts there were two probe sets present on the array platform that we used - except for the BioB gene, which was targeted with three probe sets. This results in nine probesets for which we could evaluate the effect of hybridization onto different slides. The average Pearson correlation between the 12 microarray experiments of the signals from these nine probesets was observed to be very high (97.09%) and ranged between 93.76% and 99.99%.

### Analysis of expression data

Updated transcript definitions can improve both the precision and accuracy of microarray data [76]. We used chip description files [77] downloaded from BRAINARRAY (version 11; http://brainarray.mbni.med.umich.edu). All data processing and analysis was conducted using *R* [78]. The 430 2.0 array was designed from the laboratory mouse genome, which is primarily derived from *M. domesticus* [54]. We used two approaches to help avoid systematic errors associated with this bias. First, data analysis was performed using probe logarithmic intensity error estimation (PLIER) on the signal intensity measurements as implemented with the justPlier function in BioConductor [79]. The PLIER algorithm is a model-based signal estimator that dynamically weights the probe signal intensity data using empirical probe performance. Each of the 6,998 genes was targeted with an average of 17 probes (range: 7–108). We used the PLIER algorithm to summarize the signals of these probes in order to obtain a robust gene level expression measurement. Second, we only considered genes with significantly detectable expression in all 12 individuals. A gene was considered expressed in an individual if the perfect match signal was significantly higher than the mismatch signal (Wilcoxon signed rank tests; P*<*0.01). Expression values were then quantile normalized to facilitate comparison across chips.

The primary goals of our experiment were to identify global patterns of testis gene expression with respect to (1) evolutionary divergence between *M. musculus* and *M. domesticus* and (2) divergence between sterile and fertile mice. To identify expression differences between species (*DxD* versus *MxM*), we first identified all genes with significantly different expression between groups based on gene-by-gene Student's t-tests (P<0.05), excluding genes with no variation between individuals. We then estimated the t-test p-value corresponding to an FDR of 5%, as implemented with fdrtool [80], to evaluate the robustness of all global patterns inferred from this pairwise contrast to multiple comparisons. Next we employed a hierarchical approach to define a conservative set of sterility-correlated genes. We first identified all genes with significantly different expression between groups based on gene-by-gene Student's t-tests (P<0.05) in each of the three possible pairwise comparisons between fertile genotypes (*MxM*, *DxD*, *DxM*) and the *MxD* sterile F_1_ hybrid mice. To estimate the FDR of these individual pairwise contrasts, we performed all ten possible sample-label permutations of each pairwise comparison to derive an empirical distribution of significant outcomes under the null hypothesis of no differences between the groups [81]. The FDR was then calculated as the ratio of the median number of significant outcomes in our permutations to the observed number of significant outcomes at a 5% cutoff. The estimated FDR's for individual pairwise comparisons in this study (3.2%–19.3%; see Results) are comparable to those in other studies [82], [83] and indicate that the results of the individual pairwise contrasts are not dominated by type I error. Nonetheless, because of the potential for false discovery of individual genes, we emphasize global patterns of expression difference with respect to genomic location rather than focusing on individual genes. To further reduce the FDR, we also restricted our focus to genes that were significantly different between the reciprocal hybrids and at least one of the parental lines. While direct estimation of the FDR for this hierarchically-defined set is complicated by non-independence of partially overlapping comparisons, this set of genes should be much more conservative than the three individual pairwise comparisons with respect to false positives associated with male sterility. Finally, we repeated all analyses using more stringent definitions of sterility-correlated and a more conservative threshold for our gene-by-gene t-tests (P<0.01, estimated FDR 0.5–7.5%) and observed the same global patterns with respect to expression divergence on the X chromosome.

To evaluate our data in the context of up- versus down-regulation of genes in sterile males we binned sterility-correlated genes into three groups: genes with higher mean expression in sterile *MxD* mice versus the mean expression of each of the three fertile genotypes, genes with lower expression in sterile mice, or genes where sterile mice showed intermediate levels of expression.

### Gene set enrichment analysis

To determine if differentially expressed genes were randomly distributed across the genome we performed chromosome-wise hypergeometric tests with Bonferroni correction for multiple hypothesis testing. Gene annotation was based on Ensembl version 52 of NCBI build 37 of the mouse genome. Of the 6,998 expressed genes in our analysis, 6,882 were annotated as protein-coding genes, 13 as pseudogenes, two as retrotransposed genes, and one small nucleolar RNA.

We used the GermOnline Systems database [84] to associate genes with particular testis cell types. These cell type associations derive from a series of microarray experiments on enriched cell populations [53] and denote in which testis cell population [somatic (Sertoli cells), mitotic (spermatogonia), meiotic (spermatocytes), or postmeiotic (round spermatids) cells] a given gene showed the greatest level of induction in and is not necessarily indicative of cell type specific expression. We also used additional expression data [39] to provide a second, more detailed account of cell type specific expression patterns on the X chromosome. Using microarray analysis of enriched cell populations, X-linked genes were classified into five expression groups: group A - expressed in mitotic cells (A and B spermatogonia) and repressed in meiotic (pachytene spermatocytes) and postmeiotic cells (round spermatids); group B - expressed in mitotic cells, repressed in meiotic cells, and expressed in postmeiotic cells; group C - expressed in postmeiotic cells; group D - variable expression; group E - repressed in all cells. Genes with variable expression (group D) comprise a very small subset of X-linked genes [39] and were not included in our analysis. Bonferroni-corrected binomial tests were used to determine if subsets of genes were randomly distributed with respect to cell types. Because gene expression on X chromosome is non-random with respect to cell type (Table S2), expectations were generated independently for the X chromosome and the autosomes and were based on the observed distributions of the total number of expressed genes in a given cell type.

### Molecular evolutionary analysis

We analyzed all one-to-one orthologs between mouse and rat using Ensembl annotation version 48 (www.ensembl.org; NCBI mouse build 37). Rates of protein evolution were calculated based on the number of nonsynonymous substitutions per nonsynonymous site (*dN*) normalized by the number of synonymous substitutions per synonymous site (*dS*), as previously reported [85].

### Genotyping of *Prdm9*

The critical region of *Prdm9* occurs in the C terminus, and the sterility phenotype correlates with alternative numbers of C_2_H_2_ repeats [50]. This region was targeted using published primers [50] centered around chromosome 17 position 15,249,000 (NCBI m36 mouse genome assembly). Ten pmol of each primer was combined with 5 nmol dNTP, 50 nmol MgCl_2_, BioRad Platinum taq polymerase, buffer, and water to 25 µL, and run for 35 cycles of: 94 C 20 sec, 57.5 C 20 sec, 68 C 90 sec. The classical inbred strains C57BL/6J (sterile allele, 12 C_2_H_2_ repeats) and C3H (fertile allele, 13 C_2_H_2_ repeats) were included as controls. PCR products were scored on a 2% agarose gel.

### Data deposition

The expression data reported in this paper have been deposited in the NCBI Gene Expression Omnibus (GSE17684).

## Supporting Information

Figure S1 Chromosomal distribution of sterility-correlated genes. The observed versus expected distribution of the 902 sterility-correlated genes is given for each chromosome. Only the X chromosome (red) showed a significant deviation (Bonferroni-corrected P<0.0001; 81 observed versus 27.3 expected) based on chromosome-wise hypergeometric tests.
(0.14 MB TIF)

Figure S2 Overlap and chromosomal distribution of expression differences between sterile and fertile mice based on pairwise t-tests (P<0.01). (A) The Venn diagram gives the numbers of genes with significantly different expression for the three pairwise contrasts between sterile *MxD* mice and the three fertile mouse genotypes (*DxM*, *MxM*, *DxD*). The estimated FDR for each comparison is given in parentheses and was determined with permutation. There were 226 genes that were significantly different between the reciprocal hybrids and at least one of the parental lines (gray shading). (B) The observed versus expected chromosomal distribution of the 226 sterility-correlated genes. Only the X chromosome (red) showed a significant deviation (Bonferroni-corrected P≪0.0001; 46 observed versus 6.8 expected) based on chromosome-wise hypergeometric tests.
(0.32 MB TIF)

Figure S3 X chromosome expression in fertile *MxM* and sterile *MxD* hybrid mice across spermatogenic cell types. The number of genes with higher average expression in *MxD* versus *MxM* males across four general patterns of X-linked spermatogenic expression in mice [30] (see text for details). Genes with variable expression (group D) were not included in this analysis. M =  mitotic expression, PM =  postmeiotic expression. (*) Denotes a significant deviation from the binomial expectation of equal proportions (all P<0.0001). For comparison, the same contrast is provided for all expressed autosomal genes.
(0.12 MB TIF)

Table S1 Expression data for all 6,998 testis expressed genes.
(2.98 MB XLS)

Table S2 Non-random cell type distribution of X-linked genes expressed during spermatogenesis. The X chromosome shows a significant excess of mitotic genes and no meiotic genes.
(0.03 MB DOC)

Table S3 Forty-five sterility-correlated genes.
(0.04 MB XLS)
