# Supplementary figures and images for: Widespread Over-Expression of the X Chromosome in Sterile F1 Hybrid Mice
Source: PLoS Genet. 2010 Sep 30;6(9):e1001148. doi: 10.1371/journal.pgen.1001148 (PMC2947990; doi:10.1371/journal.pgen.1001148)

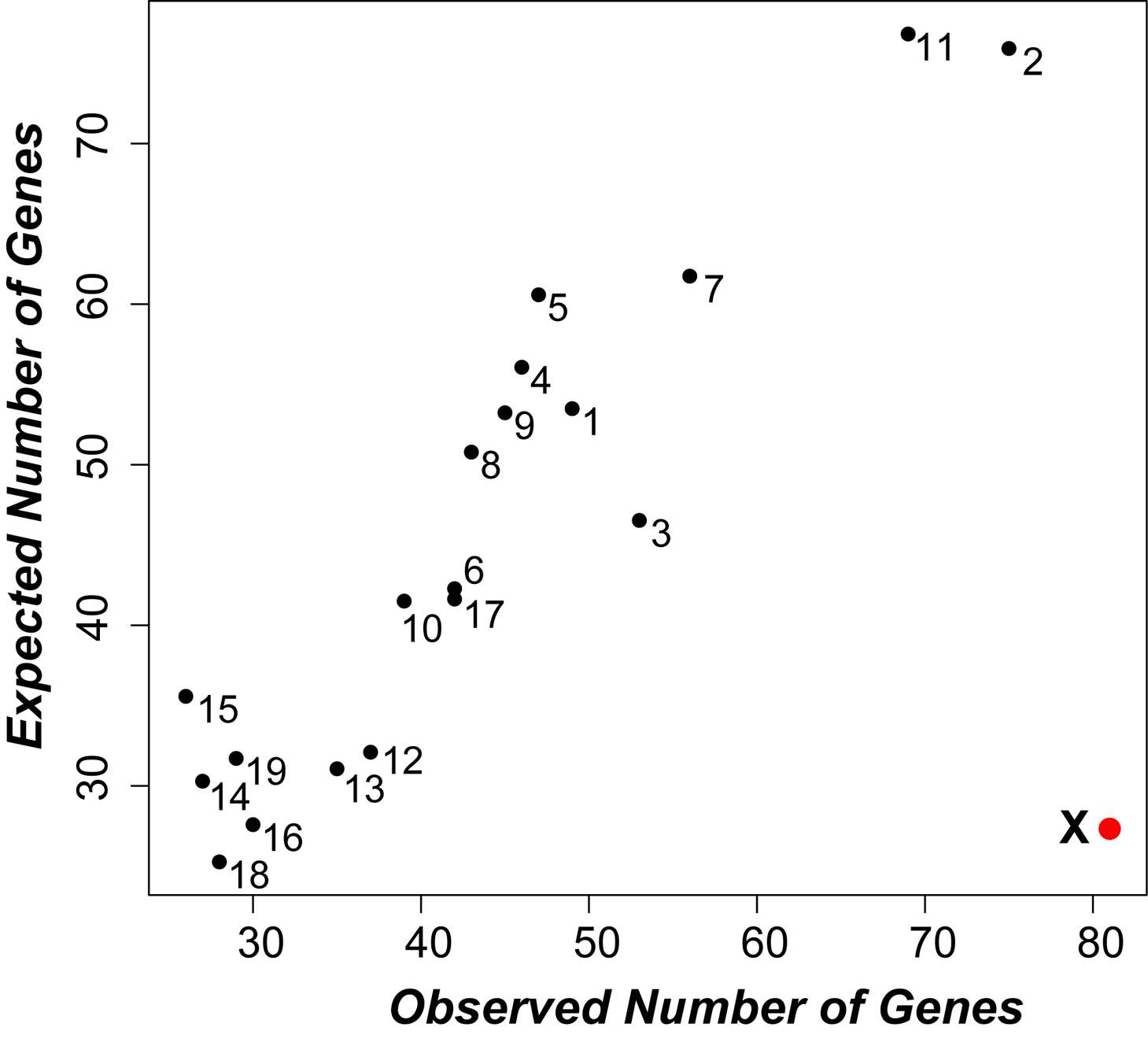

Supplement: Figure S1 — Chromosomal distribution of sterility-correlated genes. The observed versus expected distribution of the 902 sterility-correlated genes is given for each chromosome. Only the X chromosome (red) showed a significant deviation (Bonferroni-corrected P<0.0001; 81 observed versus 27.3 expected) based on chromosome-wise hypergeometric tests. (0.14 MB TIF) [file pgen.1001148.s001.tif]

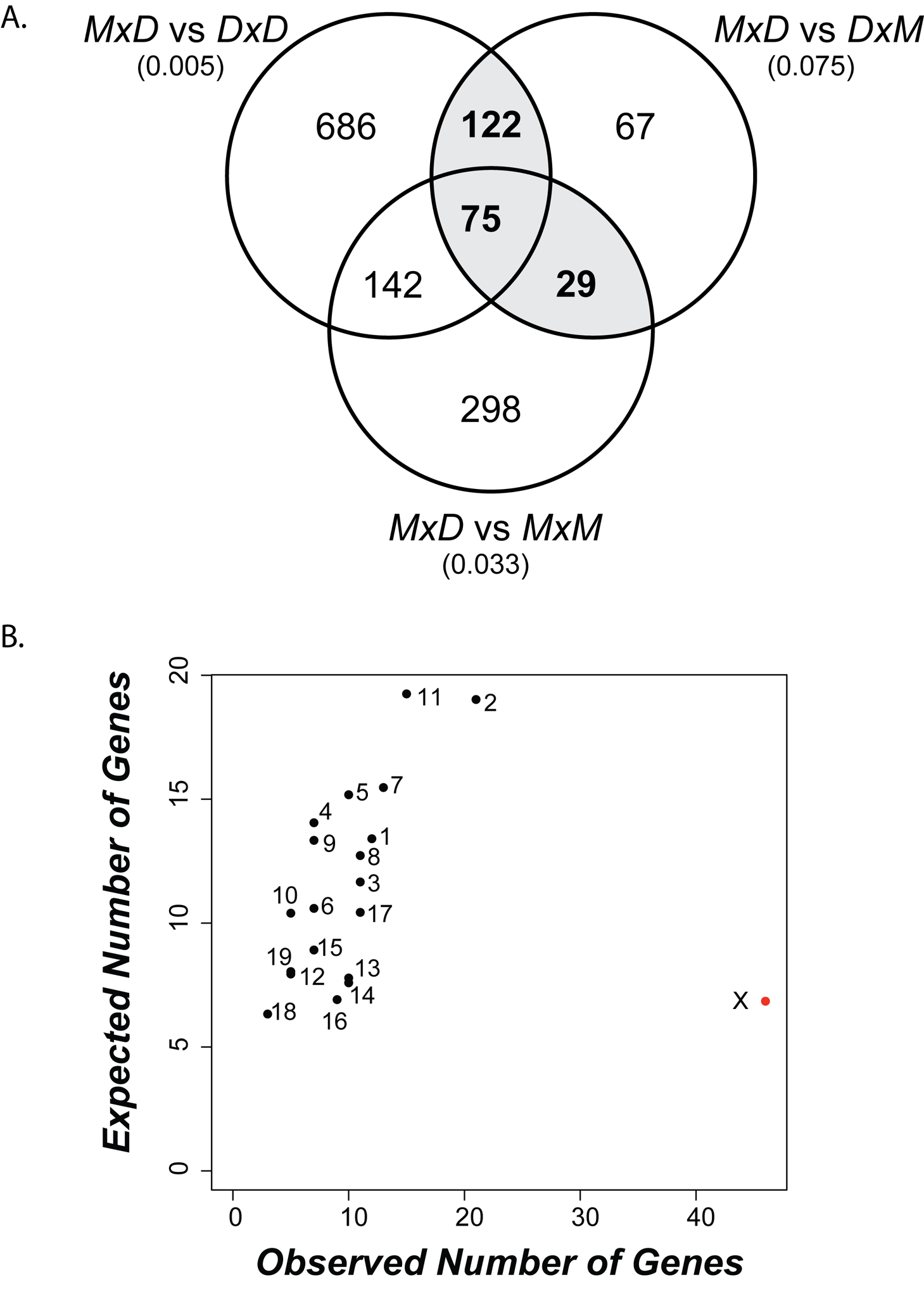

Supplement: Figure S2 — Overlap and chromosomal distribution of expression differences between sterile and fertile mice based on pairwise t-tests (P<0.01). (A) The Venn diagram gives the numbers of genes with significantly different expression for the three pairwise contrasts between sterile MxD mice and the three fertile mouse genotypes (DxM, MxM, DxD). The estimated FDR for each comparison is given in parentheses and was determined with permutation. There were 226 genes that were significantly different between the reciprocal hybrids and at least one of the parental lines (gray shading). (B) The observed versus expected chromosomal distribution of the 226 sterility-correlated genes. Only the X chromosome (red) showed a significant deviation (Bonferroni-corrected P≪0.0001; 46 observed versus 6.8 expected) based on chromosome-wise hypergeometric tests. (0.32 MB TIF) [file pgen.1001148.s002.tif]

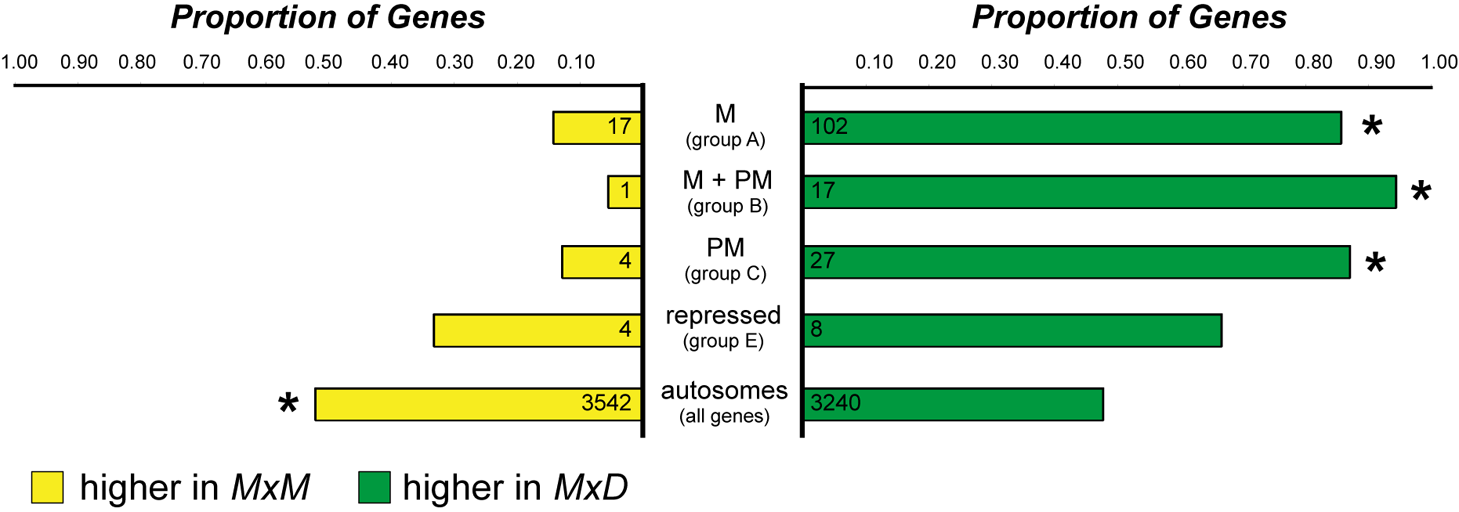

Supplement: Figure S3 — X chromosome expression in fertile MxM and sterile MxD hybrid mice across spermatogenic cell types. The number of genes with higher average expression in MxD versus MxM males across four general patterns of X-linked spermatogenic expression in mice [30] (see text for details). Genes with variable expression (group D) were not included in this analysis. M = mitotic expression, PM = postmeiotic expression. (*) Denotes a significant deviation from the binomial expectation of equal proportions (all P<0.0001). For comparison, the same contrast is provided for all expressed autosomal genes. (0.12 MB TIF) [file pgen.1001148.s003.tif]
