# Supplementary material for: Widespread Over-Expression of the X Chromosome in Sterile F1 Hybrid Mice
Source: PLoS Genet. 2010 Sep 30;6(9):e1001148. doi: 10.1371/journal.pgen.1001148 (PMC2947990; doi:10.1371/journal.pgen.1001148)
Supplement: Table S2 — Non-random cell type distribution of X-linked genes expressed during spermatogenesis. The X chromosome shows a significant excess of mitotic genes and no meiotic genes. (0.03 MB DOC) [file pgen.1001148.s005.doc]

**Table S2**. Non-random cell type distribution of X-linked genes expressed during spermatogenesis. The X chromosome shows a significant excess of mitotic genes and no meiotic genes.

|  | Observed^1^ | Expected^2^ | P |
| --- | --- | --- | --- |
| Somatic | 31 | 21.8 | 0.1582 |
| Mitotic | 94 | 56.7 | <0.0001 |
| Meiotic | 0 | 55.4 | <0.0001 |
| Postmeiotic | 48 | 39.0 | 0.4857 |

^1^Represents a subset of the 212 X-linked genes expressed in testis that could be associated with particular cell types.

^2^Expectations were generated based on the observed distributions of expressed genes in a given cell type and tested with a Bonferroni-corrected binomial distribution.
